# Review: Neurological Complications From Therapies for Pediatric Brain Tumors

**DOI:** 10.3389/fonc.2022.853034

**Published:** 2022-04-11

**Authors:** Thien Nguyen, Sabine Mueller, Fatema Malbari

**Affiliations:** ^1^ Department of Pediatrics, University of San Francisco, San Francisco, CA, United States; ^2^ Department of Neurology, Neurosurgery and Pediatrics, University of San Francisco, San Francisco, CA, United States; ^3^ Division of Neurology, Department of Pediatrics, Baylor College of Medicine, Houston, TX, United States

**Keywords:** neurological complications, brain, cancer, therapeutics, pediatrics

## Abstract

Surgery, chemotherapy and radiation have been the mainstay of pediatric brain tumor treatment over the past decades. Recently, new treatment modalities have emerged for the management of pediatric brain tumors. These therapies range from novel radiotherapy techniques and targeted immunotherapies to checkpoint inhibitors and T cell transfer therapies. These treatments are currently investigated with the goal of improving survival and decreasing morbidity. However, compared to traditional therapies, these novel modalities are not as well elucidated and similarly has the potential to cause significant short and long-term sequelae, impacting quality of life. Treatment complications are commonly mediated through direct drug toxicity or vascular, infectious, or autoimmune mechanisms, ranging from immune effector cell associated neurotoxicity syndrome with CART-cells to neuropathy with checkpoint inhibitors. Addressing treatment-induced complications is the focus of new trials, specifically improving neurocognitive outcomes. The aim of this review is to explore the pathophysiology underlying treatment related neurologic side effects, highlight associated complications, and describe the future direction of brain tumor protocols. Increasing awareness of these neurologic complications from novel therapies underscores the need for quality-of-life metrics and considerations in clinical trials to decrease associated treatment-induced morbidity.

## Introduction

Advances in therapeutic modalities, stemming from broader understanding of biologic pathways of cancer, ranging from targeted molecular treatments to viral-based therapies, have demonstrated and continue to show great potential for improving pediatric neuro-oncologic outcomes. As survivorship for pediatric brain tumor patients continues to improve, the corresponding increase in morbidity from treatment-associated complications are a growing and significant concern. Patients who undergo craniospinal radiation are at a significant risk of secondary malignancies, especially those with an underlying defect in tumor suppressor genes, such as neurofibromatosis type 1 and 2 and Li Fraumeni syndrome. Compared to their healthy peers and survivors of non-central nervous system (CNS) cancers, survivors of brain tumors experience the lowest physical, psychological, and social health outcomes (1–3). With these developing concerns, ongoing trials aim to address the management of therapy-induced complications. To further understand the landscape of brain tumor therapies, we reviewed traditional and novel treatment modalities of CNS tumors, explore their underlying molecular mechanism of action, highlight associated complications, and outline the future direction of brain tumor treatment protocols.

## Traditional Therapies

### Chemotherapy

Chemotherapy, in addition to surgery and radiation therapy have been the mainstay of pediatric brain tumor treatment over the past decades. In addition to profound pancytopenia, chemotherapies can cause neurologic deficits, including encephalopathy, ataxia and motor weakness (4). The most used chemotherapeutic agents for brain tumors include vincristine, vinblastine, carboplatin, cyclophosphamide, cisplatin, ifosfamide, and etoposide, of which the latter two are most associated with encephalopathy (incidence of 10-40%) (4–7). Vincristine and vinblastine can rarely cause syndrome of inappropriate antidiuretic hormone secretion (SIADH) which can subsequently lead to seizures. Neuropathy from vinka alkaloids is a more common adverse effect (16-78%) (5, 8). Cisplatin and carboplatin are ototoxic drugs, which can cause permanent high-frequenting hearing loss (42-71%) and consequently severe learning and developmental impairments in young children (5, 9–14). Among patients, who received cisplatin and carboplatin therapy for brain tumors, 4 patients with glioma (57%), 15 with medulloblastoma (88%) and 3 with primitive neuroectodermal tumor (50%) had ototoxicity. However, sodium thiosulfate has demonstrated otoprotective effects with lower rates of hearing loss for children with hepatoblastoma, who underwent cisplatin treatment in a randomized phase III trial (15). In this study of 109 children, patients who received sodium thiosulfate had a 48% lower incidence of hearing loss (RR= 0.52; 95% CI, 0.33 to 0.81; P = 0.002) compared to those who did not receive sodium thiosulfate, with no impact on overall survival (15). A similar phase III trial (NCT00716976) investigating the use of sodium thiosulfate for cisplatin-associated hearing loss in brain tumors, specifically medulloblastoma, neuroblastoma and germ cell tumors has recently been completed with results pending.

Chemotherapeutic agents, such as vincristine, cyclophosphamide, ifosfamide, etoposide, gemcitabine, and cisplatin can result in blood pressure elevations, which may cause posterior reversible encephalopathy syndrome (1-10%), presenting with headaches, seizures, vomiting visual changes, and altered mental status (16, 17). Generally, management of these chemotherapy associated complications is supportive care. Intolerable neurologic side effects can often be managed by dose reduction or modifying the treatment regimen (18). **Table 1** summarizes neurologic side effects of commonly used chemotherapeutic agents for the treatment of brain tumors.

**Table 1:** Common chemotherapy for brain tumors by mechanism of action and side effects.

Common Chemotherapies	Neurological side effects	Duration of side effects
***Alkylating Agents***		
Cyclophosphamide (Cytoxan)	Encephalopathy, seizures, vision changes, RCVS	Within 24 hours of administration, symptoms are reversible
Ifosfamide	Acute neurotoxicity with disorientation, lethargy, somnolence, hallucinations, coma, and seizures.	Within 24 hours to 6 days after therapy, reversible but can have long lasting neurologic impairment
Busulfan	Dizziness, headaches, seizures	Seizures may occur within 2 days of therapy
Temozolomide (TMZ)	Headaches, Worsening of focal neurologic symptoms (increased intracranial pressure), increased permeability of blood brain barrier.	Occurs at initiation of therapy, but self-limited
Nitrosureas: Carmustine (BCNU) > Lomustine (CCNU)	Confusion, fatigue, ataxia, seizures, coma, focal neurologic weakness, stroke, optic neuropathy. Rarely can cause necrotizing leukoencephalopathy	Occurs at time of initiation up to 6 months
Platinum-based: cisplatin > carboplatin > oxaliplatin	SIADH, ataxia, motor weakness, urinary retention, myopathy, ototoxicity, ageusia, sensory neuropathy	Peripheral neuropathy: Occurs within 2-6 months of treatment. Dose dependent toxicity and neuropathy may worsen after use. May resolve 6-8 months after treatment though may still have residual symptoms
	Cisplatin: stroke, confusion, seizures, motor impairment	
	Oxaliplatin: allodynia with cold	
***Antimetabolites***		
Methotrexate	Headache, meningitis, temporary neurologic deficits, seizures, learning disorder, memory impairment, gait disturbance, bowel/urinary incontinence, posterior reversible encephalopathy syndrome, transverse myelitis, cytotoxic edema	Transverse myelitis: occurs 2 days- 2 weeks following intrathecal administration, irreversible and contraindication to give intrathecal methotrexate
		Subacute neurotoxicity (stroke like events): occurs 2-6 days post-infusion and usually self resolves afterwards
		Leukoencephalopathy: occurs 2 weeks-months of first dose
***Topoisomerase Inhibitors***		
Camptothecans: topotecan, irinotecan	Sleep difficulties, vertigo	Occurs at initiation of therapy, but self-resolves afterwards
Etoposide (VP-16)	Seizures, altered mental status, headaches	Occurs during and around drug infusion, but self resolves afterwards
***Alkaloids***		
Vinca Alkaloids: Vincristine, Vinblastine	SIADH, seizures, peripheral sensorimotor neuropathy of the long nerves, cranial and autonomic neuropathies, encephalopathy, ataxia, hallucinations, parkinsonism, self-limited cortical blindness	Peripheral neuropathy: occurs within 2 months of therapy and can worsen with continued infusions. In pediatric patients, neuropathy can persist long-term and present later.
***Others***		
Retinoids (all-trans retinoic acid)	Elevated intracranial pressure, headaches, ataxia, changes in color vision, oculogyric crisis (rare)	Self-resolves after administration

RCVS, reversible cerebral vasoconstriction syndrome; SIADH, Syndrome of inappropriate antidiuretic hormone secretion. '>' refers to severity of side effects for specific agents in the same chemotherapy class.

### Radiation Therapy (RT)

Of the RT modalities, fractionated RT is commonly used in pediatric brain tumors, because it delivers a lower dose per fraction, while increasing the probability of interrupting tumor cells during times of cell division and radiosensitivity, sparing normal tissue (19, 20). Radiation dose and volume correlate with functional outcomes, especially in the pediatric population. The extent of RT (focal vs. craniospinal) depends on the brain tumor pathology with certain brain tumors, such as medulloblastoma, receiving craniospinal RT due to the higher risk of CNS metastasis.

RT can cause both short and long-term neurotoxicity. While short-term neurotoxicity presents as pseudoprogression with worsening of acute neurologic symptoms, somnolence syndrome, radiation necrosis, long-term toxicity can include neurocognitive impairment, vasculopathy, and secondary malignancies like high grade gliomas and meningiomas (21). Proton therapy, a newer radiotherapeutic modality has been successful in improving cognitive outcomes but has not shown benefit with any other RT-associated complications.

#### Cognitive Dysfunction

Decline in cognitive ability, including attention, memory, and information processing have profound influences on pediatric patients’ long-term health-related quality of life as it impairs job and education prospects, as well as hinders social interactions (22, 23). Children are most vulnerable to RT-associated cognitive complications with the greatest risk during infancy to early childhood. Not surprisingly, radiation dose and field and photon therapy have the most impact on neurocognitive outcomes with pediatric patients treated with craniospinal irradiation associated with the poorest cognitive outcomes compared to those receiving focal radiation only (24). The prevalence of cognitive dysfunction ranges from 40-100% for pediatric brain tumor survivors (25–27). In respect to time course, cognitive complications may occur shortly after RT but also persist years after treatment. Within the first 3-12 weeks, post radiation somnolence syndrome can occur, which presents with fatigue, nausea, vomiting fever, dysphagia, and ataxia. Though post radiation somnolence syndrome can last several weeks, they are typically self-limiting (28). RT-cognitive dysfunction occurs frequently after treatment, is associated with younger age and can be very challenging to manage (29). Pediatric patients who are treated with RT should undergo yearly neuropsychologic assessments to ensure normal development and additional educational and school support as needed.

Metformin has the potential to improve cognitive recovery in pediatric patients post-RT (30). Metformin acts on the neural stem cells in the subventricular zone and the dentate gyrus, restoring neurogenesis after RT and in turn has been associated with improvement in working memory performance in mice models (30). In a randomized placebo controlled early phase clinical trial with pediatric patients, metformin enhanced auditory-verbal recall and working memory, both associated with neurogenesis in the dentate gyrus of the hippocampus (31, 32). Side effects from metformin were safe and tolerable, including mild gastrointestinal distress, and diarrhea, consistent with its known adverse effects. Overall, metformin shows promise as a safe therapy for RT-induced cognitive dysfunction, but larger clinical trials are required before widespread adoption (30).

Due to negative impacts of quality of life from neurocognitive decline, several clinical trials have sought to proactively address and improve neurocognitive outcomes in pediatric patients post- RT through cognitive training or stimulant medications. The Children’s Oncology Group (COG) ACCL10P1 (NCT01503086) employs a computerized cognitive training program for children, who recently completed cranial RT, in efforts to prevent memory and attention dysfunction. In this study, pediatric patients engage with interactive computerized educational modules, targeting verbal working memory and visual-special skills 3-5 times a week for several weeks. COG’s ACCL0922 (NCT01381718) investigates modafinil’s role in improving attention, executive function and fatigue compared to placebo in pediatric patients, who previously underwent treatment for their primary brain tumor. Most recently, COG is conducting a Phase 3 randomized, placebo-controlled trial evaluating memantine for neurocognitive protection in children receiving cranial radiation therapy for their primary CNS tumors, ACCL2031 (NCT04939597). Though the results of these studies are pending, these trials refocus efforts towards enhancing not only survival, but also improving quality of life.

#### Vascular Complications

There are both short and long-term vascular complications that arise following RT therapy. While cerebral microbleeds can be detected as early as eight months after RT, cavernous malformations and atherosclerosis can present decades later (33, 34). Cerebral microbleeds are RT induced small vessel vasculopathies that are associated with poorer executive function, working memory and cognitive decline in pediatric patients (35–37). Patients who are treated at a younger age, with higher RT doses, and a larger treatment volume are at greater risk of developing cerebral microbleeds (38–40).

Though the mechanism of these RT-associated vascular complications remains unclear, it is thought that radiation causes hyperplasia to the endothelium and fibrinoid necrosis of the vascular walls, resulting in vessel stenosis (41–43). The resulting impaired flow and increased venous pressure can lead to migraines, Moyamoya syndrome (3%), cavernoma formation (50%) and ischemic stroke (1-12%) (43–51). Post-radiation arteriopathy increases the risk of strokes due to stenosis of the distal internal carotid arteries and proximal cerebral arteries with significant neurovascular risk in pediatric patients who receive RT adjacent to the circle of Willis (4). The Childhood Cancer Survivor Study (CCSS) revealed that pediatric brain tumor survivors, who completed therapy 5 years or longer, had a significantly elevated risk of late-occurring stroke (RR = 29; 95% CI, 13.8 to 60.6; P <.0001) compared to the sibling control group (52). The cranial RT dose was directly correlated with late-onset stroke with doses greater than 30 gray (Gy) associated with an increased risk, and the highest risk of stroke noted in RT doses above 50 Gy (52, 53). In the CCSS group, for patients who received greater than 50 Gy, the risk of stroke continued to rise throughout adulthood with the cumulative incidence of 1.1% at 10 years post diagnosis and increasing more than tenfold to 12% at 30 years (53). This increased risk over time may be due to accelerated intracranial atherosclerosis following RT as atherosclerotic risk factors of hypertension and diabetes both separately and in combination were significant predictors of stroke in the CCSS cohort (53). Due to this progressive stroke risk and modifiable risk factors, the Children’s Oncology Group recommends serial monitoring for strokes at clinic visits, routine screening for hypertension, blood glucose and lipid panel every two years in children with an elevated BMI, and yearly imaging with MRI and MRA as indicated for possible intervention, such as aspirin and surgical revascularization.

Other vascular malformations, such as cerebral cavernous malformations can occur on average 10 years post-RT and present with seizures or hemorrhage. Although there is a relatively low incidence of cavernomas 10 years post-RT, around 3-4%, patients with medulloblastoma are at a significantly higher risk (34, 43, 54). Surgical intervention may be required if there are concerns for rupture and hemorrhage (45).

Though not clearly a vascular complication, stroke-like migraine attacks (SMART) syndrome is a late-onset sequalae of radiation, which can occur 1-40 years post-RT (49). SMART syndrome presents with symptoms of repetitive migraine headaches, aphasia, persistent seizures, and other focal neurologic deficits, spanning days to weeks (49). Symptoms typically self-resolve for more than 80% of patients, but some may experience permanent neurologic deficits (55). Though the underlying etiology of SMART syndrome is not clearly defined, it is postulated that hypoperfusion and cerebrovascular changes observed on imaging may be contributing factors (56, 57). Treatment involves symptom management.

#### Radiation Necrosis

RT is thought to induce radiation necrosis *via* two mechanisms: (1) vascular injury from upregulation of pro-inflammatory markers [Vascular endothelial growth factor (VEGF), Intercellular Adhesion Molecule 1 (ICAM-1), and Tumor Necrosis Factor alpha (TNF-alpha)] and activation of sphingomyelinase and ceramide, which results in endothelial cell death, (2) reactive astrogliosis, creating pro-inflammatory environment and demyelination (58). Typically, radiation necrosis is observed within the first two years of RT but can occur as early as 6 months. However, early tumor recurrence can be often radiographically indistinguishable from radiation necrosis in high grade tumors.

While asymptomatic radiation necrosis can be managed with close observation, patients with symptomatic radiation necrosis causing focal neurologic deficits, mass effect and elevated intracranial pressure, should be initiated on corticosteroids as first-line therapy (59, 60). For patients requiring longer term treatment, bevacizumab (an anti-vascular endothelial growth factor (VEGF) monoclonal antibody) has demonstrated robust clinical and radiographic response in multicenter prospective trials, avoiding steroid-treatment toxicity (61, 62). Bevacizumab has also shown significant improvement in neurocognitive outcomes, including attention, language, concentration, memory, visuospatial orientation, and calculation at two months compared to patients treated with corticosteroids only (63). However, as an anti-angiogenesis agent, bevacizumab decreases wound healing and predisposes patients to hemorrhage and thrombosis. Aside from bevacizumab, hyperbaric oxygen therapy has also been explored but the benefit remains controversial (64). Despite proton therapy’s ability to deliver radiation more precisely, it has not been demonstrated to reduce the risk of radiation necrosis. For patients with severe symptoms and/or treatment-refractory radiation necrosis, including persistent neurologic deficits and mass effect, surgical intervention could be considered (65).

## Novel Therapies

The increasing need for targeted therapies is multifactorial and includes the need to improve outcomes where chemotherapy and radiation have failed. Concurrently, advances in molecular and gene profiling of brain tumors have enhanced our ability to diagnose, risk stratify and identify key aberrant genetic pathways, paving the way for new tailored modalities (62). However, breakthrough for novel therapies has been limited to low grade glioma and a small number of specific high-grade gliomas, namely mismatch repair-deficient glioblastoma (GBM). These new therapies can be categorized into two main approaches: (1) targeting gene mutations and dysregulated pathways involved in cellular growth differentiation, and apoptosis, such as with Mitogen-activated protein kinase kinase (MEK) and B-Raf proto-oncogene (BRAF) inhibitors, and small molecule inhibitors, and (2) upregulating immunosurveillance and immune system activation, such as through checkpoint inhibitors, chimeric antigen receptor T-cells (CAR T-cells), vaccine therapy and virotherapies.

These new modalities face a myriad of extrinsic, intrinsic and tumor-associated limitations (66, 67). Extrinsic factors consist of physical and biological barriers preventing trafficking of therapies into the intracranial space, such as the blood brain barrier and tumor microenvironment (65). For immunotherapies like CAR T-cell and virotherapies, intrinsic limitations such as limited targetable tumor antigens, insufficient proliferation or expansion and lack of durable response can also hinder clinical efficacy (63). Brain tumors, especially gliomas, can circumvent the binding and targeting of these novel therapies by upregulating expression of inhibitory ligands and cytokines, and downregulating targeted tumor antigens, preventing recognition by CAR T-cells, viral vectors, and avoiding check point inhibition (68, 69).

### Targeted Therapy

#### Mitogen-Activated Protein Kinase Kinase (MEK) Inhibitors

Aberrant mitogen-activated protein kinase (MAPK) pathway signaling is the most prevalent genetic abnormality underlying low grade gliomas in pediatric patients (70–72). In phase II clinical trials of pediatric patients, MEK inhibitors have demonstrated efficacy in pretreated, non- neurofibromatosis type 1 (NF1), recurrent optic pathway and hypothalamic low-grade gliomas, as well as BRAF-mutated or NF1- associated low grade gliomas (73, 74). MEK inhibitors can be considered as an alternative treatment modality for children with multiple lesions or have underlying mutations in BRAF and NF1 as they can avoid the paradoxical MAPK pathway activation observed when low-grade gliomas with aberrant BRAF-KIAA1549 mutations are treated with BRAF inhibitors (75). Phase 3 COG trials are currently underway to directly compare standard chemotherapy versus MEK inhibitors in NF1 associated and sporadic low-grade gliomas in the upfront as well as recurrent setting. From phase I/II trials of pediatric patients, MEK inhibitors have tolerable neurotoxicity with the most common neurologic symptom being headache (30%) and myopathy (73, 74, 76). Cobimetinib was more frequently associated with myopathy compared to other MEK inhibitors (77). Dose reduction and/or discontinuation resolved all acute and severe toxicities. These acute complications are similar to what is observed in the adult population. However, the long-term effects of continued MEK inhibition are not fully elucidated in the pediatric population. *In vivo* models of juvenile rats have demonstrated decreased bone length and delayed sexual maturation with prolonged treatment with MEK inhibitors (78). However, these changes were dose-dependent decreases in body weight, food consumption and long bone growth and consequently, the decrease of bone growth were likely related to overall growth and not due to a bone specific effect of MEK inhibition.

#### B-Raf Proto-Oncogene (BRAF) Inhibitors

The BRAF protein is a serine/threonine kinase protein, which modulates activity downstream of the Ras-Raf-MEK extracellular signal-regulated kinase (ERK) and MAPK pathway (79). Both signaling pathways play integral roles in regulating cellular differentiation, growth, development, and apoptosis. The most common mutation in the BRAF gene involves a missense mutation in the activation segment on exon 15, codon 600, resulting in a valine to glutamic acid substitution (V600E) (79, 80). This subsequent BRAF-V600E mutation is most commonly observed in gangliogliomas, papillary craniopharyngiomas, pleomorphic xanthoastrocytomas, and epithelioid glioblastomas (81). In patients with pilocytic astrocytomas, fusion of KIAA1549-BRAF gene is more often noted (81).

For brain tumors that harbor the BRAF V600E mutation, direct inhibition of this oncogenic kinase and upstream MAPK proteins can provide targeted therapy as demonstrated in a phase I/II study (82). BRAF V600E inhibitors are well tolerated with no significant neurotoxicities (83). Non-CNS related complications included pyrexia, fatigue, rash, arthralgias, arterial hypertension, decrease in left ventricular ejection fracture, and QT prolongation (83, 84). These side effects are directly correlated to the drug dosage and improved with alternation/reduction of medication. Like MEK inhibitors, the acute complications from BRAF inhibition are similar between adult and pediatric population. Long-term, the use of BRAF V600E inhibitors can lead to squamous cell carcinoma and keratoacanthoma, which may be attributed to paradoxical activation of MAP kinase in cells lacking the BRAF mutation (83).

Though BRAF V600E monotherapy can be effective for a period of time, tumors typically progress in a matter of months (85). To prevent tumor progression, concurrent inhibition of both BRAF and MEK has demonstrated efficacy in pre-clinical models. A recent phase II trial for high- and low-grade gliomas has illustrated a clinical response with combination dabrafenib, a BRAF inhibitor, and trametinib, a MEK inhibitor (86, 87). In this trial, common neurological complications included fatigue, and headache, consistent with prior side effects seen with monotherapy. However, there is an increased risk of cardiovascular adverse events, including pulmonary embolisms, arterial hypertension and left ventricular ejection fracture with dual BRAF and MEK inhibitor therapy compared to BRAF monotherapy (84). As with MEK inhibitors, cessation and dose reduction of these drugs decreased toxicity, but also decreased efficacy.

#### Small Molecule Inhibitors

Small molecule inhibitors target the tumor microenvironment, as well as tumor-dependent pathways involved with cell growth, invasion and angiogenesis. Because of their small size, they can readily pass through the blood brain barrier to reach CNS tumors. There are several small molecule inhibitors currently studied in clinical trials, including inhibitors of vascular endothelial growth factor (VEGF), cyclin dependent kinase (CDK) 4/6, and histone deacetylase pathways, as well as Onc 201, a dopamine receptor D2 (DRD2) antagonist.

The class of VEGF inhibitors prevent angiogenesis (88). VEGF modulates vasculature formation and permeability, crucial for tumor infiltration, growth, and metastasis (89). Several VEGF inhibitors can also block platelet derived growth factor receptors, another angiogenesis pathway and c-kit signaling, involved with driving CNS tumor growth (90–92). In phase I/II trials for pediatric patients with brain tumors, who underwent VEGF inhibitor therapy, the following neurologic side effects were reported: intracranial hemorrhage (8-10%), lower extremity pain (8%), dizziness (6%), headache (5-15%), muscle weakness (5%), myalgia (4%), altered consciousness (4%), dysphagia (2-11%), reversible posterior leukoencephalopathy syndrome (2%), seizures (2%), hydrocephalus (2%), peripheral motor neuropathy (2%), blurry vision (2%), leukoencephalopathy (2%), papilledema (2%), and fecal incontinence (2%) (93–96). Of note, because neovascularization is critical for physiologic skeletal develop, these children also had notable growth plate abnormalities (9%), which may severely impact growth, adult height, and skeletal maturation (97). Overall, when compared to children with solid tumors who underwent treatment with VEGF inhibitors, there were significantly fewer neurologic side effects, with the most common being pain (10%), suggesting that these neurologic adverse events may be intrinsic to CNS tumors and related to disease progression (90, 91, 98).

CDK are vital regulators of cell cycle progression with CDK4 and CDK6 as integral components (99). The overexpression of CDK4 and 6 are linked to tumor invasion and tumorigenesis in several cancer phenotypes (100). CDK 4/6 inhibitors prevent downstream phosphorylation of retinoblastoma, arresting cell division at the G1 check point. From phase I/II trials for pediatric patients with progressive brain tumors, CDK 4/6 inhibitors were well tolerated with neurotoxicities including: ataxia (24%), dizziness (23%), seizure (21%), headache (19-31%), gait disturbance (14%), and muscle weakness (14%) (101, 102). Of note, seizures were only seen in the heavily pre-treated patients, defined as those who underwent more than 4 prior interventions, such as RT and/or myeloablative chemotherapy/biologics and/or myeloablative chemotherapy with bone marrow stem cell rescue (101).

Histone deacetylase inhibitors are epigenetic regulators that block histone deacetyltransferase (HDAC) activity (103). HDAC condense chromatin by removing acetyl groups from histones, which can repress gene transcription and activity, enabling overexpression of genes involved with proliferation, while downregulating genes involved with differentiation and tumor regulation. HDAC inhibitors regulate gene expression not only through blockade of HDAC, but also by regulating aberrant chromatin structures, which can alter cell migration, differentiation, growth, and angiogenesis (104, 105). In phase I/II trials with HDAC inhibitors for all tumors types, notable neurologic side effects include: lower extremity myopathy (33%), hydrocephalus (17%), pyramidal tract syndrome (17%), seizures (17%), ataxia (17%), Bell’s palsy (17%), muscle weakness (17%), nystagmus (17%), blurry vision (17%), dysgeusia (17-28%), dizziness (15%), headache (6-33%) and neuropathy (6%) (106–110). There were no improvements in either progression or overall survival in these early phase trials.

Onc201 is a small molecule in the imipridone class, which can bypass the BBB, and acts through several mechanisms for anti-tumor effect. Originally, Onc201 was discovered as a downstream regulator of the TNF-related apoptosis-inducing ligand (TRAIL) gene transcription pathway (111). Because immune cells express TRAIL receptors on their surface, Onc201 can selectively induce apoptosis, activating the body’s anti-tumor immunosurveillance without damaging normal cells. Subsequent work has identified underlying molecular targets, including selective competitive and non-competitive antagonism of DRD2 and allosteric activation of caseinolytic protease P (ClpP), both of which are overexpressed in multiple malignancies and can serve as specific anti-tumor therapeutic targets (112–115).

Phase I/II trials with Onc201 have demonstrated tolerable neurotoxicity, with no patients requiring dose adjustment or drug discontinuation for adverse effects (116, 117). In a Phase II recurrent glioblastoma trial, the most common side effect likely attributable to weekly Onc201 was dizziness (5%) (116). While other neurological adverse events were also reported in the trial, including headache (50%), memory impairment (20%), seizure (20%), and paresthesia (15%), these symptoms were attributed to disease progression and not Onc201. Additional potential neurologic complications reported include tinnitus, dysgeusia, altered mental status and gait instability (116–118). Because of the limited data of adverse side effects, and unique mechanism of action, several open trials have explored the potential for synergistic benefit of Onc201 in conjunction with RT, chemotherapy, checkpoint inhibitors and targeted agents to potentiate therapy response and target separate mechanisms of resistance (118). Thus far, pre-clinical *in vivo* data has yielded promising responses though clinical trial results have yet to be reported (111, 119).

### Immunotherapy

#### Check Point Inhibitors

Recently, check point inhibitors have gained traction in pediatric trials given their efficacy across different cancers. However, they have shown limited efficacy in brain tumors, except in hypermutant glioblastoma with mismatch repair deficiency (120). Ordinarily, T-cells interact with antigen presenting cell’s major histocompatibility complex receptors through programmed death 1 (PD-1) and its associated programed cell death ligand 1/2 (PD-L1, PDL-2), as well as cytotoxic T lymphocyte antigen 4 (CTLA-4) receptors to modulate self-immunity and immune tolerance. PD-1 inhibits T cell activity through interaction with PD-L1 and PD-L2 (121). Tumor cells can evade immunodetection by up-regulating immune checkpoint expression of these negative regulators PD-1 and CTLA-4 and in effect desensitize and exhaust T-cells from recognizing the tumor (122). By blocking the tumor’s PD-L1/PDL-2 binding sites to the T-cell, these checkpoint inhibitors allow for persistent T-cell activation, immunosurveillance and recognition of the tumor (123–125). However, these uninhibited T-cells also have capacity to target normal tissue and result in adverse autoimmune-associated complications (125).

The timing of checkpoint inhibitors may influence the anti-tumor immune response for brain tumor patients. In a randomized multi-institution clinical trial, patients who received both neoadjuvant and adjuvant pembrolizumab (an anti-PD-1 monoclonal antibody) after surgical resection showed improved overall and progression free survival compared to those who received adjuvant therapy alone (126). This clinical response can be attributed to neoadjuvant inhibition of PD-1, resulting in upregulation of interferon response by tumor infiltrating lymphocytes in the tumor microenvironment (126). In turn, inhibition of tumor cell proliferation and cell cycle function occurs. Patients who received only adjuvant PD-1 blockade did not demonstrate similar T-cell upregulation, interferon gene expression, or inhibition of tumor cell proliferation (126).

Though the specific mechanism underlying autoimmune complications from check point inhibitors are unclear, it is thought to be mediated through aberrant T-regulatory cell function from up-regulation of Th-1 and Th-17 activity, and in turn cytokine production, such as IL-6 and IL-17 (124, 125, 127). In addition, enhanced autoantibody production following PD-1 and CTLA-4 inhibition can contribute to cross-reactivity of antigens found on both tumors and normal nervous system tissue, resulting in non-specific targeting (125). This enables these antibodies to bind and impair muscles, nerves, and neuromuscular junctions. Because of this molecular mimicry and widespread involvement of the nervous system, checkpoint inhibitor therapy can induce a significant autoimmune response, which can include: fatigue, headache, inflammation of the pituitary gland, CNS vasculitis, aseptic meningitis, myositis, retinopathy, posterior reversible encephalopathy, auto-immune mediated disorders (myasthenia gravis, cerebellar degeneration, enteric neuropathy, polyradiculopathy, vascular neuropathy, autoimmune encephalitis, progression of demyelinating diseases) (4, 128).

Current management of neurologic complications from check point inhibitors depends on the severity of neurologic deficits, associated patient comorbidities, and risks/benefits of initiating immunotherapy. Though there are no current guidelines, corticosteroids, IVIG, and plasmapheresis are considered first line management strategies. If these initial therapies are unsuccessful, immunosuppressants, such as: rituximab, mycophenolate, cyclophosphamide, and methotrexate can be used (125, 129). For refractory cases, proteasome inhibitors (bortezomib), IL-17 blockers and tacrolimus have also been trialed (130). The decision to discontinuing check point inhibitors in the setting of treatable auto-immune related neurologic complications is controversial. Resumption of checkpoint therapy is dependent on various factors including life expectancy, cancer progression, severity of initial immune-associated neurologic events, and the oncologist’s own comfort with management (125).

#### Chimeric Antigen Receptor T-Cells (CAR T-Cell)

The initial success with CD19 targeted CAR T-cells for CNS leukemia garnered attention for the potential for immunotherapy for CNS tumors (131). Recent trials have targeted common antigens against epidermal growth factor receptor variant III (EGFRvIII), GD2 and HER2 found in pediatric CNS tumors (132, 133). However, one of the unique difficulties with immunotherapy for CNS lesions, involves managing the potential tumor inflammation associated neurotoxicity, which can be fatal (134).

Three sequelae of CAR T cell treatment include: cytokine release syndrome (CRS), immune effector cell-associated neurotoxicity syndrome (ICANS) or CAR T cell neurotoxicity, and recently tumor inflammation associated neurotoxicity (TIAN), which is specifically observed in CNS tumors. CRS typically occurs the first week of therapy and initially presents with fever, but can develop with symptoms of tachycardia, hypotension and multi-organ dysregulation, including liver failure and disseminated intravascular coagulation (135). Depending on the treatment approach, symptoms can resolve within 2 weeks (136). CRS is mediated through macrophage activation and subsequent cytokine release by monocytes, macrophages and CAR T-cells (137).

Compared to CRS, ICANS occurs 2-11 days after therapy, presenting with tremor, lethargy, cognitive, motor and speech disturbances and in severe cases focal neurologic deficits, such as seizures, cerebral edema, increased intracranial pressure, severe ataxia, and loss of consciousness. Unfortunately, patients may experience chronic neurotoxicity, lasting over several months (138). ICANS can occur independently of CRS; however, more severe CRS symptoms, as well as higher serum CAR T-cells, and elevated CSF protein, leukocytes, interferon-gamma, IL-6 and IL-10, are correlated with the presence of neurotoxicity (138, 139). Though the exact mechanism of these neurologic complications is poorly understood, *in vivo* models show pronounced meningeal inflammation on histopathology in mice with ICANs compared to those without neurotoxicity (140).

Uniquely observed in brain tumors, TIAN occurs due to intratumoral inflammation (141). As a result of tumor inflammation, patients can experience worsening neurologic deficits and concerns for increased ICP, hydrocephalus and herniation. There remains a paucity of literature and experience with TIAN, but in one single case, management was centered on CSF diversion for elevated intracranial pressure (141).

Generally, the treatment for CRS involves supportive measures as these adverse events are self-limited but can be lethal. Initiation of treatment will depend on the grade/severity of CRS. Since these complications are likely mediated through the release of cytokines, including IL-6, IL-1, and GM-CSF, tocilizumab, an IL-6 inhibitor can be used to treat severe CRS without affecting the immune response (136, 142). Unlike for CRS, tocilizumab has no effect on ICANS, due to difficulty passing the BBB. In a similar mechanism, anakinra, an IL-1 receptor antagonist, has demonstrated efficacy in murine models with reversing neurotoxicity, not observed with IL-6 blockade, as well as, in a case series treating high grade ICANs, and achieving clinical response in half of patients (143, 144). However, further data is required before anakinra can be adopted for ICANS therapy. Steroids can be used for ICANS though it can decrease the efficacy of CAR T-cell therapy (145). In practice, steroids are reserved for severe CRS refractory to tocilizumab treatment and for patients presenting with ICANS.

#### Vaccine Therapies

Several phase I/II clinical trials have demonstrated increased overall survival with active immunotherapy using autologous dendritic cell (DC) vaccines to target glioblastoma and high-grade gliomas (146–153). As antigen presenting cells, DC can activate and maintain primary immune responses when paired with tumor associated antigens. After exposure to tumor antigens, DC phagocytize and express them as cell surface receptors for CD4+ and CD8+ lymphocyte activation. Similar to CAR T-cells, which require autologous T-cell collection, DC are harvested from the patient, cultured and pulsed with tumor associated antigens before being transfused back to the patient to promote antigen-specific activation of T-cells (154).

In addition to DC vaccines, RNA vaccines have emerged as a promising therapy due to their lower cost, rapid development, and high potency. RNA vaccines can activate both the humoral and cell-mediated immune response as it can deliver different tumor specific antigens and can encode entire tumor antigens, activating a more robust T-cell response (155). Because nucleic acid vaccines also lack viral or protein-derived products during manufacturing, these RNA based therapies are well tolerated with lower cross reactivity (156). One emerging trial from the Pacific Pediatric Neuro-oncology Consortium trial 20 (PNOC020) utilizes an RNA-lipid particle vaccine to treat high grade gliomas in children and adults.

From recent trials, there have been limited neurotoxicities following DC or RNA vaccine administration, with reports of increased seizures and radiographic pseudoprogression in a small subset. In adult patients, who underwent DC vaccine therapy for their high-grade gliomas, a small cohort of patients had increased seizure frequency (5-10%) (157–159). In pediatric patients with recently diagnosed high grade glioma or diffuse midline glioma, after administration of the peptide vaccine with glioma-associated antigen epitopes, radiographic evidence of pseudoprogression (19%) was noted, with corresponding symptoms of ataxia, cranial neuropathies and hyperventilation (153). Dexamethasone improved neurologic symptoms from pseudoprogression. The most common complications were typical for routine vaccines, including fatigue, myalgia, headaches, fevers and localized rashes at the injection site (150, 160–162). The incidence of these complications was not significantly different between the treatment and control group. Lymphopenia was the most serious adverse event for patients with high grade gliomas, who received the vaccinations (149). In all trials, there were no deaths were attributable to the DC or peptide vaccination.

Aside from the complications of the vaccine, the optimal injection method has not been clearly delineated, as many different routes have been described, including subcutaneous, intraperitoneal, intranodal, and intrathecal (160). Overall, vaccine therapies are an emerging class of therapeutics that may be efficacious against gliomas with tolerable adverse effects.

#### Virotherapy

Like vaccine therapy, virotherapy elicits an inflammatory response and induces tumoricidal effects to overcome glioma’s immunosuppressive microenvironment, but through viral vectors, such as herpes, coxsackie, parvovirus, poliovirus, cytomegalovirus, and adenovirus (163–165). Currently two classes of virotherapies have been used: (1) replication-competent oncolytic viruses, and (2) gene therapy viral vectors, both which require direct injection into the tumor or resection cavity.

Oncolytic viruses are genetically modified to specifically recognize and infect tumor cells, while avoiding normal cells. Following replication within the cancer cell, these oncolytic viruses can induce apoptosis, triggering the release of additional viral progeny and tumor antigen, which activates a robust innate and adaptive immune response (166, 167). Conversely, gene therapy viral vectors induce immune response by inserting desired genomic information into the tumor cell’s transcription and translation mechanism and in turn manufacture new proteins, such as toxic byproducts or cytokines to trigger an anti-tumor response (168). With new advances in viral vectors, current virotherapies now encompass components of both oncolytic and gene-therapy viral properties to enhance therapeutic effects, such as with DNX-2440 and Toca 511. DNX-2440 was designed as an oncolytic virus, but can also transduce *X40L*, a gene which activates immune response in tumors to augment the anti-tumor response (14). Toca 511, a gene therapy virus with the ability to replicate, can continue to proliferate and infect tumor cells even after the initial treatment (169).

From initial studies for the treatment of recurrent high-grade gliomas, virotherapy has been well tolerated though this in part depends on the specific viral vector (170). In one study of 61 adult glioblastoma patients with non-pathogenic polio virus-rhinovirus vaccine through intra-tumoral infusion, most patients experienced at minimum one neurologic side effect though over 66% were grade 1 (171). These adverse events included: headache (52%), hemiparesis (50%), seizures (45%), dysphagia (28%), cognitive changes (25%), visual field deficits (19%), paresthesia (13%), gait abnormalities (10%), dystonia (2%), and facial weakness (2%) (171). There were three severe adverse events, which ranged from grade 4 cerebral edema (2%) to grade 5 intracranial hemorrhage (2%) and grade 5 seizure (2%).

Toxicities from other early clinical trials have been primarily associated with viral infection and replication, including fever, lymphocyte depletion and malaise (168). These systemic responses were mild and self-limited. The most serious complications noted in those trials was also encephalopathy and cerebral edema though again patient sample size is limited (172). Although there are theoretical concerns of off-receptor viral targeting or uncontrolled viral replication, studies have failed to demonstrate any evidence of neurovirulence. For virotherapy-induced neurotoxicity, bevacizumab was used during these trials to manage tumor associated edema. Other symptoms of viral malaise and fever were managed with supportive care (171).

## Future of CNS Therapies to Reduce Morbidity and Mortality

With the implementation of new treatment modalities for brain tumors, therapy-associated morbidity should also be considered. To address therapy complications, we need to transition to create protocols that both enhance survival and decrease morbidity yet maintain efficacy. The goals of reducing chemo- and radiation neurotoxicity have been accomplished through de-escalation of therapy and revision of protocols for brain tumors with good prognosis, as well as targeted drug therapies. For example, patients with medulloblastoma, WNT subtype, have a more favorable prognosis and are therefore being treated, in the setting of clinical trials, with de-escalation of therapy, including reduction of the radiation and chemotherapy doses. In similar efforts, protocols for germinomas, which are generally radio-sensitive and with low mortality, have been modified to incorporate neoadjuvant chemotherapy followed by a reduced RT dose to alleviate long-term radiation-associated neurotoxicity (173). In children with low grade gliomas, treatment with targeted therapies have higher response rates than chemotherapy. However, there are concerns for significant toxicity. Phase III clinical trials are underway comparing chemotherapy versus targeted therapy, which should provide additional information on response and toxicity (NCT04166409).

In addition to de-escalation of therapy or using targeted therapy, other trials are investigating neuroprotective medications. For example, COG clinical trials are exploring sodium thiosulfate for otoprotection in patients with medulloblastoma and memantine for preventing cognitive dysfunction in children receiving cranial radiation. Ultimately, the goal of brain tumor treatment is transitioning towards increasing long term survivorship without impacting patients’ quality of life.

## Conclusion

As new promising therapies emerge for pediatric patients with brain tumors, long-term morbidity must also be considered and factored into clinical trial design. The ultimate goal for future treatment should be to provide targeted, effective, and safe therapy to improve the lives of all children with brain tumors.

## Author Contributions

TN and FM wrote and revised the manuscript. SM revised and contributed through discussion to the manuscript. All authors have read and approved the manuscript.

## Conflict of Interest

The authors declare that the research was conducted in the absence of any commercial or financial relationships that could be construed as a potential conflict of interest.

## Publisher’s Note

All claims expressed in this article are solely those of the authors and do not necessarily represent those of their affiliated organizations, or those of the publisher, the editors and the reviewers. Any product that may be evaluated in this article, or claim that may be made by its manufacturer, is not guaranteed or endorsed by the publisher.
